# Supplementary material for: Minimum dietary diversity and its determinants among lactating mothers in five Sub-Saharan African countries: A multilevel analysis
Source: PLoS One. 2025 Mar 5;20(3):e0308590. doi: 10.1371/journal.pone.0308590 (PMC11882081; doi:10.1371/journal.pone.0308590)
Supplement: Attachment — Submitted filename: Response to Reviewers.docx [file pone.0308590.s004.docx]

**Response to reviewers' comments**

PONE-D-24-31238

**Title: Minimum Dietary Diversity and its Determinants among Lactating Mothers in Five Sub-Saharan African countries: A multilevel analysis**

**Journal:** PLOS ONE

**Authors:** *Alebachew Ferede Zegeye, Tadesse Tarik Tamir, Desale Bihonegn Asmamaw, Desalegn Anmut Bitew, Elsa Awoke Fentie, Bewuketu Terefe, Rahel Mulatie Anteneh, Lemlem Daniel Baffa, Misganaw Guadie Tiruneh, Kaleb Assegid Demissie, Tadele Biresaw Belachew, Wubshet Debebe Negash, Melak Jejaw*

**Subject:** Submission of revised manuscript

Yours sincerely,

**Alebachew Ferede Zegeye (on behalf of all authors)**

University of Gondar, Gondar, Ethiopia

**Response to editor comments**

1. A recurring comment has been the use of 16 food groups while 10groups are recommended. Authors should please give a justification and cite relevant literature.

- **Response**: Dear editor, Thank you for your observation regarding the use of 16 food groups instead of the 10 groups recommended. We apologize for the confusion caused by the mention of 16 food groups in our analysis. This was indeed a typographical error, and we have now corrected the text to reflect the proper use of the 10 food groups as outlined in the FAO's latest MDD-W guidelines. Kindly see the amendments in the revised manuscript on page 7, lines 185-189.

1. Authors should kindly take note and correct some errors and typos throughout the manuscript

- **Response**: Dear editor, Thank you for pointing out the need for corrections. We have thoroughly reviewed the manuscript and addressed all identified errors and typographical issues. Additionally, we conducted a meticulous proofreading process to ensure the manuscript is error-free and meets the journal's quality standards. Thank you.

2. Please ensure that your manuscript meets PLOS ONE's style requirements, including those for file naming. The PLOS ONE style templates can be found at

https://journals.plos.org/plosone/s/file?id=wjVg/PLOSOne_formatting_sample_main_body.pdf andhttps://journals.plos.org/plosone/s/file?id=ba62/PLOSOne_formatting_sample_title_authors_affiliations.pdf.

- **Response**: Dear editor, thank you for the recommendations. Thank you for your feedback. We have reviewed the PLOS ONE style requirements and ensured that the manuscript adheres to the specified formatting guidelines, including the file naming conventions. The title, authors, affiliations, and main body sections have been formatted as per the PLOS ONE templates. We have also made any necessary adjustments to ensure consistency with the style guidelines provided in the sample documents. Kindly see the amendment in our revised manuscript.

3. When completing the data availability statement of the submission form, you indicated that you will make your data available on acceptance. We strongly recommend all authors decide on a data sharing plan before acceptance, as the process can be lengthy and hold up publication timelines. Please note that, though access restrictions are acceptable now, your entire data will need to be made freely accessible if your manuscript is accepted for publication. This policy applies to all data except where public deposition would breach compliance with the protocol approved by your research ethics board. If you are unable to adhere to our open data policy, please kindly revise your statement to explain your reasoning and we will seek the editor's input on an exemption. Please be assured that, once you have provided your new statement, the assessment of your exemption will not hold up the peer review process.

- **Response**: Dear editor, Thank you for your feedback regarding the data availability statement. We have now addressed this by sharing the entire dataset as a supplementary file accompanying the manuscript. This ensures compliance with the journal's open data policy while facilitating accessibility for interested readers and researchers. We have updated the data availability statement to reflect this, confirming that the data is freely accessible alongside the publication. If further clarification or modifications are required, we are happy to address them promptly.

4. Please review your reference list to ensure that it is complete and correct. If you have cited papers that have been retracted, please include the rationale for doing so in the manuscript text, or remove these references and replace them with relevant current references. Any changes to the reference list should be mentioned in the rebuttal letter that accompanies your revised manuscript. If you need to cite a retracted article, indicate the article’s retracted status in the References list and also include a citation and full reference for the retraction notice.

- **Response**: Dear editor, our reference list has been reviewed to ensure completeness and accuracy. No retracted articles have been cited in the manuscript. All references listed are current and valid sources that have been properly vetted for scientific rigor and relevance to the study. We have replaced older references with more recent publications where applicable, ensuring up-to-date information is presented. Any necessary changes to the reference list has outlined in the rebuttal letter accompanying our revised manuscript. Thank you for bringing this to our attention.

**Response to reviewer comments**

**Reviewer #1**

- **Major Comments**

* **Abstract**: The abstract provides a concise summary of the study, covering the background, methods, results, and conclusions. However, the following improvements could be made

- Include the actual coverage of Minimum Dietary Diversity (MDD) with confidence intervals.
- **Response:** Dear reviewer, First and foremost, thank you for your insightful comments. We have included the actual coverage of the Minimum Dietary Diversity (MDD) with confidence intervals on page 2, lines 40-41, in the revised manuscript.
- While key findings are reported, the specific categories of the association between certain factors and dietary diversity could be more clearly articulated. For example, it is unclear how the distance to a health facility affects dietary diversity (DD). Does it refer to a long or short distance, or a significant or minor problem? In addition, when comparing secondary or higher education with no formal education, the reference group is incorrectly identified, as there is another level: primary education. Clarify if the place of delivery refers to home or another location.
- **Response:** Dear reviewer, Thank you for your feedback. In response to your concern about the clarity of the associations between certain factors and dietary diversity, we have revised the manuscript to better articulate these relationships. Specifically, we clarified the impact of distance to a health facility by distinguishing between long and short distances, and emphasized whether it represents a significant or minor challenge for accessing nutrition-related services. Regarding education, we have taken the reference group for comparison, no formal education as the baseline category, rather than primary education since literatures supported. Finally, we clarified that the place of delivery refers to institutional delivery rather than home delivery, to ensure greater accuracy in our description. Kindly see the revised manuscript on page 2, lines 42-47.
- The results, such as the proportion of lactating mothers achieving dietary diversity, could benefit from contextualization. Comparing dietary diversity across countries within the abstract could better summarise national differences.
- **Response:** Dear reviewer, Thank you for the suggestion. Due to the word limit in the abstract, we have refrained from including detailed comparisons across countries. However, we have addressed this important point in the discussion section of the revised manuscript, where we provide a contextual comparison of dietary diversity across different national settings to highlight variations and their implications.

* **Introduction**: The introduction thoroughly addresses the importance of dietary diversity among lactating mothers and situates the study within a broader public health context. However, it could be improved by:

- Providing a clearer rationale for selecting the five Sub-Saharan African countries. A stronger justification for their inclusion based on specific nutritional challenges is needed. While the authors mention that women in these countries do not meet the minimum dietary diversity, this does not sufficiently justify the selection of these countries over others in Sub-Saharan Africa that face similar issues.
- **Response:** Dear reviewer, we appreciate your insightful comment. The selection of the five Sub-Saharan African countries was primarily guided by the availability of data on maternal nutrition in the DHS datasets, which is critical for determining our outcome variable, minimum dietary diversity. Specifically, these countries used DHS Guide 8, which includes comprehensive indicators related to maternal nutrition, enabling us to assess dietary diversity accurately. In contrast, other countries in the Sub-Saharan Africa rely on DHS Guide 7, which does not incorporate these essential variables. Therefore, our choice was based on the availability the relevant data related to maternal nutrition to achieve the study's objectives.
- There is limited discussion of gaps in the existing literature on multilevel determinants. Highlighting where this study contributes new insights would better position its relevance. Pooling data alone does not offer an opportunity to address country-specific determinants, particularly since the included countries are not representative of the entire Sub-Saharan African region or any specific sub-region of the continent.
- **Response:** Dear reviewer, we appreciate your observation and have revised the discussion to address the identified gaps. While it is true that the included countries are not fully representative of the entire Sub-Saharan African region, our study contributes new insights by leveraging pooled data to overcome the limitations of smaller, institution-based pocket studies with restricted sample sizes. By analyzing multilevel determinants at both the individual and community levels, this study provides a broader understanding of factors influencing minimum dietary diversity among lactating mothers, which previous research has often overlooked. Additionally, we acknowledge the importance of country-specific determinants and have emphasized that while our findings offer valuable regional insights, they also highlight the need for future studies to explore these dynamics in more diverse sub-regional contexts to strengthen policy implications tailored to specific country needs.

***** Methods

The methodology is robust, utilising multilevel mixed-effects logistic regression to account for both individual and community-level factors. However, there are a few points of concern:

- The study uses DHS data from five countries, but the selection process for these countries is not fully justified. Providing a clearer explanation of why these countries were chosen over others would strengthen the credibility of the findings. Simply using the most recent datasets is insufficient justification.
- **Response:** Dear reviewer, Thank you for your in-depth review of our manuscript. The five Sub-Saharan African countries included in this study were selected based on the availability of critical data on maternal nutrition in their DHS datasets, specifically focusing on the outcome variable, minimum dietary diversity. These countries provided comprehensive maternal nutrition indicators essential for accurately assessing dietary diversity, such as detailed food group consumption data. However, many other DHS datasets lacked these essential variables, making them unsuitable for inclusion in the study. Therefore, our selection was driven by the need to ensure valid analyses rather than merely relying on the most recent datasets. Kindly see the amendments in the revised manuscript on page 6, lines 139-144.
- The term "lactating mothers" requires a clearer definition within the study.
- **Response:** Dear reviewer, Thank you for your insightful comment. The term "lactating mothers" has been clearly operationalized in the revised manuscript on page 6, lines 235–237.
- The FAO's MDD-W (latest version) includes ten distinct food groups. It is unclear how the authors arrived at 16 food groups for their analysis. Additionally, the acceptable dietary diversity cut-off is five out of ten food groups. Using the same cut-off for 16 food groups requires justification, as this results in approximately 30% coverage rather than the intended 50%.
- **Response:** Dear reviewer, Thank you for your insightful comment. We apologize for the confusion caused by the mention of 16 food groups in our analysis. This was indeed a typographical error, and we have now corrected the text to reflect the proper use of the 10 food groups as outlined in the FAO's latest MDD-W guidelines. We also acknowledge the concern regarding the acceptable dietary diversity cut-off. In line with the FAO's recommendations, we have applied the correct cut-off of five out of ten food groups for our analysis, ensuring consistency with the intended 50% coverage. We appreciate your careful review and have made the necessary revisions to ensure accuracy in the revised manuscript. Kindly see the amendments on page 7, lines 185-189.
- While the multilevel modeling approach is appropriate, the justification for selecting this method could be better explained for non-expert readers.
- **Response:** Dear reviewer, Thank you for your insightful feedback. To enhance clarity for non-expert readers, we have elaborated on the justification for using multilevel modeling in the revised manuscript. Please see the point in the revised manuscript on page 11-12, lines 285-296.

* Discussion, conclusions, and limitations

- While some comparisons are made with studies from Ethiopia, India, and Bangladesh, these are largely limited to prevalence statistics. The authors should elaborate on why dietary diversity levels might differ in these countries, considering cultural, economic, and health system differences.
- **Response:** Dear reviewer, we thank you for your constructive comments and concerns. Dear reviewer, we have addressed the differences in dietary diversity levels between Ethiopia, India, and Bangladesh in the revised manuscript, specifically in the section on page 21, lines 442-451. In this paragraph, we elaborate on the cultural, economic, and health system factors that could contribute to the variations in dietary diversity across these countries.
- The DHS data for the various countries may not have been collected in the same period. Seasonality, which affects food availability and consumption patterns, is a known factor. Comparing country-level dietary diversity without accounting for this limitation weakens the analysis.
- **Response:** Dear reviewer, we acknowledge that the data from different countries in the DHS may have been collected during varying time periods, which could introduce seasonality effects that may influence food availability and consumption patterns. However, the DHS data are representative at the national level and account for a range of socio-demographic factors, which helps mitigate the potential impact of seasonality on dietary diversity. We have acknowledged this limitation in the manuscript and suggest that future research could explore more detailed seasonal variations using data from specific months or regions to provide a more nuanced analysis. Kindly see the revised manuscript on page 24, lines 532-334.
- The policy implications of the findings could be expanded to provide specific recommendations for the five countries studied. Although the study mentions that ministries should focus on antenatal care utilisation and education, it lacks concrete policy suggestions or frameworks. For example, Ghana has existing policies to promote education, which could be better addressed.
- **Response:** Dear reviewer, we thank you for your constructive comments and concerns. We have expanded the policy implications of our findings to provide more specific recommendations for the five countries studied. In particular, we emphasize that the health ministries of Burkina Faso, Kenya, Ghana, Mozambique, and Tanzania should prioritize addressing the needs of women who underutilize antenatal care services and those without access to formal education. For example, we have highlighted existing policies aimed at promoting education and recommended leveraging these frameworks to improve access to and utilization of antenatal care, ensuring that these efforts specifically target vulnerable populations. Kindly see the amendments on page 24, lines 542-550.
- The limitations section is brief and does not mention potential issues such as recall bias in dietary data. While the recall period is 24 hours, it is still possible that mothers may forget some details. Additionally, the study's limitations should include a discussion on the interpretation and use of the MDD indicator. Although information is collected at the individual level, the indicator is more useful for population-level inferences and not for drawing individual-level conclusions. Dietary patterns can change overnight and may not reflect normal dietary habits in the household.

**Response:** Dear reviewer, we appreciate your valuable feedback. We have now expanded the limitations section to address the potential for recall bias in dietary data, acknowledging that despite the 24-hour recall period, mothers may still forget some details. Additionally, we have included a discussion on the interpretation of the Minimum Dietary Diversity (MDD) indicator, clarifying its primary utility for population-level inferences rather than individual-level conclusions. We also recognize that dietary patterns can fluctuate and may not always reflect typical household habits, and this limitation has been incorporated into the revised manuscript. Kindly see the amendments on page 24, lines 534-538.

- **Minor Issues**

 * Grammar, such as in the abstract: "variables associated to..." should be corrected to "associated with." Additionally, “Gahanna” in the results section should be corrected to “Ghana.” The use of lowercase letters to begin words in the tables (e.g., "primary education") should also be addressed.

- **Response:** Dear reviewer, Thank you for pointing out these issues. We have corrected the grammatical error in the abstract, changing 'associated to' to 'associated with.' We have also updated 'Gahanna' to 'Ghana' in the results section as suggested. Regarding the use of lowercase letters in the tables, we have revised the text to ensure consistency by capitalizing the first letters of all relevant terms, including 'Primary Education,' to align with standard formatting conventions. Kindly see the tracked change manuscript.

* The reference style does not conform to the journal’s requirements.

- **Response:** Dear reviewer, we appreciate the insightful remarks you made. We have thoroughly reviewed the journal’s reference guidelines and made the necessary corrections to ensure all citations now comply with the required format. We apologize for the oversight and appreciate your attention to this detail. The updated reference list has been revised accordingly in the manuscript. Thank you.

**Reviewer #2**

***** The study aimed to assess the minimum dietary diversity and its determinants among lactating mothers in five Sub-Saharan African (SSA) countries (Kenya, Burkina Faso, Ghana, Mozambique and Tanzania). The manuscript gives an explanation of dietary diversity and has also highlighted the health and growth impacts of inadequate dietary diversity for both the mother and infant. The study setting is also very fitting because SSA is indeed facing several challenges particularly in relation to nutrition which is adversely impacting the health and general wellbeing of women and children especially. It is imperative for contextual research like this study, to shed more light on the issue and possibly generate data that can inform the development of targeted interventions. The study identified that dietary diversity among lactating mothers in the five sampled SSA countries was low due to an inter play of some individual and community level factors.

What I like about this study is the fact that, the authors highlighted the different and several nutritional related problems existing in Tanzania, Ghana, Mozambique, Burkina Faso and Kenya. Though having similar settings, there are yet some varied and unique features of each of these countries and the authors were able to pick out the very unique nutritional deficiencies and dietary inadequacies lactating mothers and their infants encounter in these different settings, providing us with a comprehensive understanding of the state of nutrition in SSA.

I also like the fact that the study did not only focus on the individual level factors as determinants of dietary diversity but delved into community level factors as well. Under the individual level factors, I was expecting to see variables like religion and ethnicity as these variables may impact behaviors, beliefs and practices that invariably influence dietary diversity. Since agriculture plays a role in food in security, it would have been nice to capture the food system including farming activities which may vary across the countries. In all, the paper is good and I approve for its acceptance and publication once the comments raised are addressed.

- **Response:** Dear reviewer, First and foremost, thank you for your insightful constructive comments. Thank you for your valuable feedback and insightful comments on our study. Regarding the individual-level factors, we acknowledge the importance of religion and ethnicity in influencing dietary diversity; however, religion was excluded from our analysis due to multicollinearity issues, which would compromise the reliability of the results. Additionally, ethnicity is not consistently recorded in the standard DHS datasets for the included countries, limiting its inclusion in our study. We appreciate your suggestion on the role of agricultural practices and food systems and agree this is an important area for future research to further explore the contextual determinants of dietary diversity.

***** **Major Points**

1. Authors should clarify why sixteen food groups were used for assessing Minimum Dietary Diversity for Women (MDD-W). The food and Agricultural organization (FAO) recommend 10 food groups: i.e., grains, white roots and tubers and plantains, dark green leafy vegetables, meat, poultry and fish, pulses, nuts and seeds, vitamin A rich fruits and vegetables, other fruits, other vegetables, eggs and milk and milk products (FAO, 2021). Some food groups can be sub-divided to ensure clarity but at the point of calculating the dietary diversity scores, the sub groups are not included in the computation (FAO, 2021).

- **Response:** Dear reviewer, Thank you for your insightful comment. We apologize for the confusion caused by the mention of 16 food groups in our analysis. This was indeed a typographical error, and we have corrected the text to reflect the proper use of the 10 food groups as outlined in the FAO's latest MDD-W guidelines. While our analysis adheres to the FAO's 2021 MDD-W guidelines, the food groupings in the DHS dataset differ slightly in presentation. Specifically, the DHS records dietary intake as "cereals, white roots, tubers, and plantains; dark green leafy vegetables; other vitamin A-rich vegetables and fruits; other vegetables; other fruits; animal-source foods (meat and poultry, fish and seafood, eggs, milk, and dairy products); legumes, nuts, and seeds; oils and fats; sweets; and beverages, condiments, and miscellaneous." For our analysis, we grouped the food items into the FAO's 10 recommended categories during computation of dietary diversity scores, ensuring consistency with the MDD-W guidelines. We have revised the manuscript to clarify this on page 7, lines 185–189. Thank you.

2. The recommended cut-off score by FAO is ≥ 5 food groups for adequate diversity and < 5 food groups for inadequate. Could Authors explain the choice cut off score used in the manuscript?

- **Response:** Dear reviewer, we appreciate for raising this important point. We utilized the FAO's recommended cut-off of ≥5 food groups for adequate dietary diversity and <5 food groups for inadequate dietary diversity. This categorization is based on the FAO's Minimum Dietary Diversity for Women (MDD-W) guidelines, which identify consumption of at least five out of ten defined food groups as indicative of adequate dietary diversity among women of reproductive age, including lactating mothers. Therefore, our cut-off score of ≥5 food groups aligns with the established global standard, ensuring comparability of our findings with other studies using this metric. Kindly see the revised manuscript on page 8, lines 189–192. Thank you.

***** **Minor points**

1. Background, line 4, the word “of” has been omitted. Please correct.

- **Response:** Dear reviewer, we greatly appreciate your in-depth review of our manuscript. We have carefully reviewed the text and corrected the omission of the word "of" to ensure clarity and grammatical accuracy. The revised sentence now reads: "Dietary Diversity (DD) among lactating mothers is the practice of consuming a wide range of foods across different food groups to meet the nutritional needs of their health and that of their breastfeeding child. We appreciate your attention to detail.

2. Methodology, population and eligibility criteria: The age range mentioned there is “15-59 years”. The results section however indicated “15-49 years”. Please reconcile.

- **Response**: Dear reviewer, we thank you for your constructive comments and concerns. Thank you for pointing out the discrepancy in the age range. This was a typographical error. The maternal age range in the DHS dataset is indeed 15-49 years, and we have now corrected this throughout the revised manuscript for consistency. We appreciate your attention to detail.

3. Methods: The “of” has been omitted from the first sentence “to assess the variation in minimum dietary diversity lactating mothers”.

- **Response:** Comment accepted and amended accordingly. Dear reviewer, we would like to appreciate your in-depth review of our manuscript. The omission of “of” in the sentence “to assess the variation in minimum dietary diversity lactating mothers” has been corrected in the revised manuscript on page 12, line 303.

4. Under random effects in the methods please delete repeated word.

- **Response**: Dear reviewer, we thank you for your valuable recommendations. Dear reviewer, we have carefully revised the methods section to remove the redundancy and ensure clarity. Specifically, the repetition of "in" in the sentence "the variation or heterogeneity in in minimum dietary diversity" has been corrected. Additionally, the overall section has been reviewed for grammatical consistency and streamlined for improved readability without altering the substantive content. Kindly see the revised manuscript of the random effects section.

5. Double check and clarify the spelling “Gahanna”.

- **Response**: Dear reviewer, Thank you for pointing out the inconsistency in spelling. We have reviewed the manuscript thoroughly and identified instances where “Gahanna” was used incorrectly instead of “Ghana.” We have corrected this typographical error throughout the revised manuscript to ensure consistency and accuracy.
